# Coronary angiography-derived microcirculatory resistance predicts adverse cardiovascular events in elderly with unstable angina post-percutaneous coronary intervention

**DOI:** 10.3389/fragi.2026.1839323

**Published:** 2026-06-08

**Authors:** Kangming Li, Lele Wang, Panqing Liao, Ya’nan Hu, Liming Xiong

**Affiliations:** 1 Department of Emergency, Guiling People’s Hospital, Guilin, China; 2 Guilin Medical University, Guilin, China; 3 Department of Cardiology, The Second Affiliated Hospital of Xuzhou Medical University, Xuzhou, China

**Keywords:** coronary angiography, coronary microcirculatory dysfunction, elderly, index of microvascular resistance, unstable angina

## Abstract

**Background:**

Coronary microvascular dysfunction (CMD) has been increasingly recognized as a critical determinant of adverse cardiovascular outcomes. The coronary angiography-derived index of microcirculatory resistance (caIMR) has emerged as a promising noninvasive tool for CMD assessment, yet its prognostic value in elderly patients with unstable angina (UA) following percutaneous coronary intervention (PCI) remains insufficiently explored.

**Methods:**

This single-center retrospective cohort study enrolled 348 elderly UA patients (age ≥65 years) who underwent PCI between June 2022 and February 2024. Patients were stratified by post-PCI caIMR using a cutoff value of 2.50 mmHg s/cm. The primary endpoint was major adverse cardiovascular events (MACE), including myocardial infarction, stroke, heart failure, angina rehospitalization, and cardiovascular death. Multivariable Cox regression and subgroup analyses were performed to evaluate the prognostic consistency of caIMR across different patient characteristics.

**Results:**

Patients with caIMR ≥2.50 mmHg s/cm demonstrated significantly higher MACE rates. Multivariable analysis confirmed caIMR as an independent predictor, with diabetes mellitus also showing predictive value. Subgroup analyses revealed consistent prognostic value of caIMR regardless of hypertension status or diabetes.

**Conclusion:**

Noninvasive caIMR assessment effectively stratifies post-PCI risk in elderly UA patients.

## Background

Cardiovascular disease (CVD) has emerged as the leading cause of global mortality, accounting for approximately 18.6 million deaths annually (32% of total global mortality), with a persistently increasing incidence trend according to recent epidemiological data (Martin et al., 2024). Percutaneous coronary intervention (PCI) coupled with dual antiplatelet therapy has revolutionized the management of coronary artery disease (CAD), substantially reducing the incidence of acute cardiovascular events (Li et al., 2021; Hope et al., 2022; Zijing et al., 2023). Unstable angina, representing a critical transitional phase in the CAD spectrum between stable angina and acute myocardial infarction, constitutes a vital therapeutic window where evidence-based interventions demonstrate optimal clinical benefits (Liza et al., 2020). Nevertheless, elderly patients undergoing PCI continue to exhibit suboptimal long-term outcomes, with elevated risks of major adverse cardiovascular events (MACE) including restenosis, myocardial infarction, and heart failure (Julio et al., 2025). Notably, individuals aged ≥65 years represent a high-risk population for CVD due to age-related vascular pathophysiological alterations and prevalent comorbidities, resulting in significantly higher MACE incidence, mortality rates, and poorer prognosis compared to younger counterparts (Hope et al., 2022; Zijing et al., 2023; Xiao et al., 2024). These adverse outcomes not only substantially impair patients’ quality of life but also impose considerable burdens on healthcare systems (Clara et al., 2022). Consequently, identifying reliable prognostic biomarkers for risk stratification and developing personalized therapeutic strategies have become imperative clinical priorities.

Coronary microvascular dysfunction (CMD) plays a pivotal role in the pathogenesis and progression of cardiovascular diseases (Houyong et al., 2022; Paul et al., 2023). Even in the presence of patent epicardial coronary arteries, CMD can precipitate myocardial ischemia, fibrosis, and cardiac dysfunction (Xu et al., 2024). Consequently, accurate assessment of CMD is of paramount importance for prognostic prediction and optimized therapeutic decision-making (Filippo et al., 2014). However, current methodologies for assessing CMD are inherently limited by several constraints. As exemplified by fractional flow reserve (FFR) and index of microcirculatory resistance (IMR), these techniques necessitate invasive procedures requiring specialized pressure-wire systems and ancillary equipment (Ciaramella et al., 2023). Consequently, these techniques are not universally applicable, particularly in vulnerable patient populations with fragile vasculature or elderly individuals at heightened risk of procedural complications. Furthermore, the inherent limitations of these invasive modalities - including substantial procedural costs and time-intensive protocols - significantly constrain their widespread clinical implementation. Recently, coronary angiography-derived index of microcirculatory resistance (caIMR) has emerged as a promising noninvasive alternative, offering a novel paradigm for coronary microcirculation assessment (Dejan et al., 2023). A landmark study by Professor Junbo Ge’s team at Zhongshan Hospital demonstrated that an caIMR cutoff value ≥ 2.5 mmHg s/cm exhibits significant diagnostic performance for myocardial ischemia in patients with angina with non-obstructive coronary arteries (Wang et al., 2025). The distinctive advantage of caIMR lies in its ability to derive microcirculatory resistance indices from routine coronary angiography images through dedicated computational algorithms, eliminating the need for additional invasive procedures (Wang et al., 2025). This unique characteristic confers superior clinical accessibility and cost-effectiveness to caIMR, particularly in resource-constrained healthcare settings and for patient populations contraindicated for invasive assessment modalities.

Given the inherent limitations of current CMD assessment modalities and the emerging advantages of caIMR, this study was designed to investigate the predictive value of caIMR for major adverse cardiovascular events (MACE) in elderly patients with unstable angina undergoing PCI. We hypothesize that caIMR may serve as an independent prognostic biomarker, enabling clinicians to perform accurate risk stratification and subsequently tailor individualized therapeutic strategies.

## Materials and methods

### Study population

This single-center retrospective cohort study analyzed electronic medical records of UA patients admitted to the Department of Cardiology, The Second Affiliated Hospital of Xuzhou Medical University between June 2022 and February 2024.

Inclusion Criteria: (1) Hospitalized patients with clinically confirmed UA undergoing PCI; (2) Age >65 years; (3) Standard preprocedural medication including aspirin, ticagrelor, and statins; (4) Successful PCI procedure completion.

**FIGURE 1:**
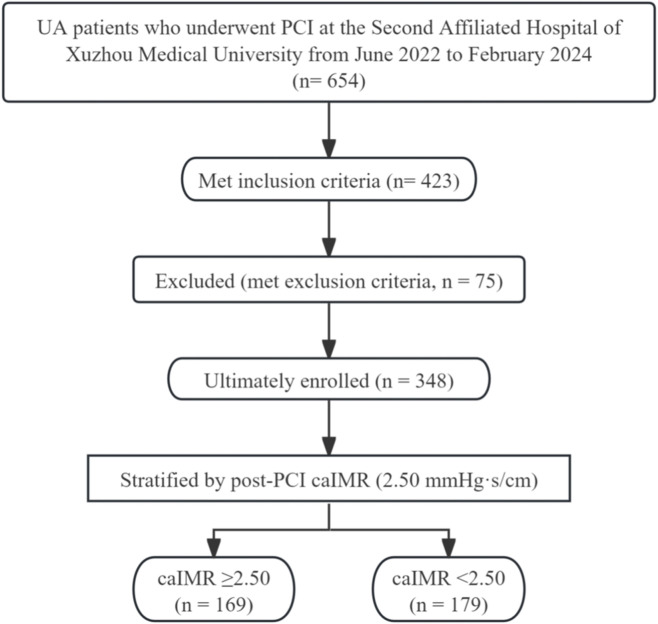
Flowchart of the study. UA, unstable angina; PCI, percutaneous coronary intervention; caIMR, coronary angiography-derived index of microcirculatory resistance.

Exclusion Criteria: (1) History of myocardial infarction or cardiomyopathy; (2) Previous coronary artery bypass grafting; (3) Thyroid dysfunction (hypo-/hyperthyroidism); (4) Comorbid conditions including liver cirrhosis, anemia, or dialysis; (5) Incomplete baseline data. A total of 654 patients with UA who were hospitalized and underwent coronary angiography during the study period. Among them, 423 met the inclusion criteria. After applying the exclusion criteria, 348 patients were ultimately enrolled in the study (Figure 1). The study protocol was approved by the Institutional Review Board of The Second Affiliated Hospital of Xuzhou Medical University (approval No. 2020120205). All patients provided written informed consent for the use of caIMR assessment during PCI, as this was an emerging technique requiring analysis of coronary angiography images. The study was conducted in strict accordance with the ethical principles of the Declaration of Helsinki.

### Data collection

We systematically collected the following clinical data through standardized electronic case report forms: (1) baseline demographics (age and sex); (2) comprehensive medical history including smoking status, alcohol consumption, history of PCI, hypertension, and diabetes mellitus (DM); (3) current cardiovascular medications (angiotensin-converting enzyme inhibitors/angiotensin receptor blockers [ACEI/ARB], calcium channel blockers [CCB], and beta-blockers [β-R]); (4) laboratory parameters comprising complete blood count (white blood cell [WBC] and hemoglobin [Hb]), glycemic control marker (fasting blood glucose [FBG] and glycated hemoglobin [HbA1c]), and full lipid profile (total cholesterol [TC], triglycerides [TG], high-density lipoprotein cholesterol [HDL-C], and low-density lipoprotein cholesterol [LDL-C]); (5) comprehensive echocardiographic assessment including left atrial anteroposterior diameter (LAAPD), left ventricular anteroposterior diameter (LVAPD), interventricular septum thickness (IVS), left ventricular posterior wall thickness (LVPW), right ventricular anteroposterior diameter (RVAPD), and left ventricular ejection fraction (LVEF); (6) Coronary angiography-derived index of microvascular resistance.

This study enrolled patients admitted between June 2022 and February 2024. Patients were prospectively followed up *via* telephone interviews at 6-month, 12-month, 18-month, 24-month, 30-month, and 36-month intervals post-discharge. The final follow-up was completed on 30 June 2025. The median follow-up duration was 27 months (IQR: 25–30). The primary outcome variable was hospitalization for MACE during follow-up, with precise readmission dates ascertained through structured patient interviews. The primary outcome variable was hospitalization for MACE during follow-up, with precise readmission dates ascertained through structured patient interviews.

### Metabolic parameter classification

Based on the lipid management standards recommended for the Chinese population in the “Chinese guidelines for lipid management (2023)”, this study conducted a detailed classification assessment of lipid levels. TC is considered ideal at <5.2 mmol/L, and elevated at ≥5.2 mmol/L; LDL-C, <2.6 mmol/L is considered ideal, 2.6–3.4 mmol/L is considered appropriate, and ≥3.4 mmol/L is considered elevated; TG are defined as <1.7 mmol/L as the normal level, 1.7–2.3 mmol/L as borderline elevated, and ≥2.3 mmol/L as elevated; HDL-C is defined as <1.0 mmol/L as a reduced level and ≥1.0 mmol/L as the ideal level (Chinese guidelines for lipid management, 2023).

According to the recommended blood glucose control levels for the elderly in China as outlined in the “Clinical guidelines for prevention and treatment of type 2 diabetes mellitus in the elderly in China (2022 edition),” fasting blood glucose is categorized as <6.1 mmol/L, 6.1–7.0 mmol/L, and ≥7.0 mmol/L, while hemoglobin A1c is categorized as <6.5 mmol/L and ≥6.5 mmol/L (Author Anonymous, 2022).

AMR was assessed using the QFR software (Shanghai Biodong Medical Technology Co., Ltd., Shanghai, China). An AMR value greater than 2.5 mmHg*s/cm reects abnormal coronary microcirculation in patients (Wang et al., 2025; Chen et al., 2023; Huang et al., 2023).

caIMR was assessed immediately following successful PCI of the target vessel, defined as restoration of TIMI ow grade 3 with residual stenosis <20%. The method for calculating single-view µQFR involved delineating the coronary artery lumen contour of the patient under examination using the QFR software. Congestive flow velocity was determined by dividing the length of the vessel centerline by the contrast filling time (Yimin et al., 2018). Subsequently, a frame with well-filled and fully exposed lumen contours was selected and the boundaries of the vessels and major side branches were outlined. The reference vessel diameter was reconstructed based on Murray’s law of bifurcation fractals, accounting for the decrease in luminal diameter at bifurcations (Tu et al., 2015). Finally, using the aforementioned hyperemic flow as the boundary condition, the pressure drop is calculated based on the fluid dynamics equation. Distal coronary pressure (Pd) was determined based on the pressure drop. µQFR was calculated as the ratio of Pd to the mean aortic pressure (Pa), whereas AMR was calculated as the ratio of Pd to the hyperemic flow velocity (V_hyp_) (Li et al., 2026).

$$\text{AMR}=\text{Pd }/V_{\text{hyp}}=\text{Pa}\times µ\text{QFR }/V_{\text{hyp}}$$

### Data analysis

All analyses were performed using SPSS 27.0 (IBM Corp., Armonk, NY, USA) and R 4.3.1 (The R Foundation for Statistical Computing, Vienna, Austria). Continuous variables were assessed for normality using the Kolmogorov-Smirnov test. Normally distributed data were expressed as mean ± standard deviation (SD) and analyzed using parametric tests, while non-normally distributed data were presented as median (interquartile range [IQR]) and analyzed using Wilcoxon rank-sum tests. Categorical variables were reported as counts (percentages) and compared using χ^2^ tests or Fisher’s exact tests, as appropriate. Variables potentially associated with the caIMR after PCI were initially screened through univariate regression analysis. Before incorporating variables with P < 0.05 in univariable analysis and the prespecified clinically relevant covariates (age, sex, LVEF, creatinine, and history of PCI) into the multivariable Cox proportional hazards regression model, we performed collinearity diagnostics using the variance inflation factor (VIF). Variables with a VIF >2 were considered to indicate significant collinearity and were subsequently excluded from the model. Subgroup analyses stratified by comorbidities and metabolic parameters were conducted to verify the robustness of findings. All tests were two-tailed, with P < 0.05 considered statistically significant.

## Result

### Comparison of clinical data between two groups

The study enrolled 348 patients with unstable angina pectoris. As demonstrated in Table 1, compared to patients with post-PCI caIMR <2.50 mmHg s/cm, those with AMR ≥2.5 mmHg s/cm exhibited significantly higher proportions of diabetes mellitus (P < 0.05) and elevated levels of FBG, HbA1c, TG, TC, and LDL-C.

**TABLE 1:** Comparison of clinical data between two groups.

Variables	Post-PCI caIMR <2.50 mmHg s/cm (n = 179)	Post-PCI caIMR≥ 2.50 mmHg·s/cm (n = 169)	P
Age, years	72 (69–78)	72 (69–79.5)	0.993
Female, n (%)	71 (39.7%)	78 (46.2%)	0.235
Medical history			
Smoking	47 (26.3%)	44 (26%)	1
Alcohol use	24 (13.4%)	26 (15.4%)	0.648
Hypertension	126 (70.4%)	113 (66.9%)	0.468
DM	42 (23.5%)	77 (45.6%)	<0.001
History of PCI	98 (54.1%)	83 (45.9%)	0.132
Cardiovascular medical therapy			
ACEI/ARB, n (%)	96 (53.6%)	75 (44.4%)	0.087
Beta blocker, n (%)	105 (58.7%)	108 (63.9%)	0.324
CCB, n (%)	135 (75.4%)	116 (68.6%)	0.188
Laboratory tests			
WBC, 10^9^/L	6.26 (4.97–7.58)	6.34 (5.28–7.81)	0.237
Hb, g/L	131 (121–142)	132 (120–144)	0.544
Creatinine, umol/L	69 (60–82)	70 (59–84)	0.791
FBG, mmol/L	​	​	<0.001
<6.1	149 (83.2%)	85 (50.3%)	​
6.1–7.0	16 (8.9%)	35 (20.7%)	​
≥7.0	14 (7.8%)	49 (29%)	​
HbA1c (%)	​	​	<0.001
<6.5	145 (81%)	85 (50.3%)	​
≥6.5	34 (19%)	84 (49.7%)	​
TG, mmol/L	​	​	<0.001
<1.7	154 (86%)	109 (64.5%)	​
1.7–2.3	19 (10.6%)	31 (18.3%)	​
≥2.3	6 (3.4%)	29 (17.2%)	​
TC, mmol/L	​	​	<0.001
<5.2	157 (87.7%)	116 (68.6%)	​
≥5.2	22 (12.3%)	53 (31.4%)	​
HDL, mmol/L	​	​	0.568
<1	55 (30.7%)	57 (33.7%)	​
≥1	124 (69.3%)	112 (66.3%)	​
LDL, mmol/L	​	​	<0.001
<2.6	147 (82.1%)	97 (57.4%)	​
2.6–3.4	18 (10.1%)	50 (29.6%)	​
≥3.4	14 (7.8%)	22 (13%)	​
Echocardiography			
LAAPD	36.69 ± 6.88	35.56 ± 7.99	0.157
LVAPD	46 (43–49)	46 (43–48)	0.928
IVS	9 (9–10)	9 (9–10)	0.793
LVPW	9 (9–10)	9 (9–10)	0.772
RVAPD	22 (21–24)	22 (20–23)	0.378
LVEF	59 (57–60)	59 (57–60)	0.142

PCI, percutaneous coronary intervention; caIMR, coronary angiography-derived index of microcirculatory resistance; DM, diabetes mellitus; ACEI/ARB, angiotensin-converting enzyme inhibitors/angiotensin receptor blockers; CCB, calcium channel blockers; β-R, beta-blockers; WBC, white blood cell; Hb, hemoglobin; FBG, fasting blood glucose; HbA1c, glycated hemoglobin; TC, total cholesterol; TG, triglycerides; HDL-C, high-density lipoprotein cholesterol; LDL-C, low-density lipoprotein cholesterol; LAAPD, left atrial anteroposterior diameter; LVAPD, left ventricular anteroposterior diameter; IVS, interventricular septum thickness; LVPW, left ventricular posterior wall thickness; RVAPD, right ventricular anteroposterior diameter; LVEF, left ventricular ejection fraction.

### Association between post-PCI AMR and MACE by Kaplan-Meier analysis

**FIGURE 2:**
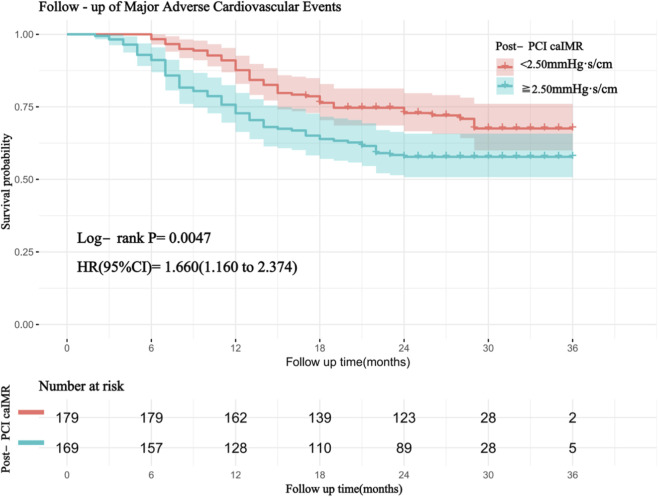
Kaplan-Meier survival curves for MACE in Elderly with UA Post-PCI with caIMR. PCI, percutaneous coronary intervention; caIMR, coronary angiography-derived index of microcirculatory resistance; MACE, major adverse cardiovascular events; HR, hazard ratio; CI, confidence interval.

Kaplan-Meier survival analysis was performed to evaluate long-term clinical outcomes. As illustrated in Figure 2, patients with post-PCI caIMR ≥2.50 mmHg s/cm exhibited significantly poorer prognosis, with a higher incidence of MACE compared to those with caIMR <2.50 mmHg s/cm (log-rank P = 0.0047; hazard ratio [HR]: 1.660, 95% confidence interval [CI]: 1.160–2.374).

### Risk factor analysis for MACE development

As presented in Table 2, univariable Cox proportional hazards regression analysis identified several significant predictors of MACE in elderly patients with UA following PCI. DM (HR = 1.681, 95% CI: 1.177–2.401; P = 0.004), elevated HBA1c levels (HR = 1.524, 95% CI: 1.064–2.183; P = 0.021), and higher caIMR (HR = 1.660, 95% CI: 1.160–2.374; P = 0.006) were significantly associated with increased MACE risk (all P < 0.05).

**TABLE 2:** Results of univariate cox regression analysis of MACE.

Variables	HR	95%CI	P
Age, years	0.990	0.960–1.020	0.501
Female, n (%)	0.935	0.654–1.338	0.714
Smoking	0.931	0.620–1.399	0.732
Alcohol use	0.861	0.509–1.457	0.578
Hypertension	0.755	0.723–1.563	0.755
DM	1.681	1.177–2.401	0.004
History of PCI	1.092	0.752–1.585	0.645
ACEI/ARB, n (%)	1.279	0.897–1.825	0.174
Beta blocker, n (%)	0.848	0.593–1.213	0.369
CCB, n (%)	0.846	0.563–1.271	0.421
WBC, 109/L	0.996	0.978–1.015	0.674
Hb, g/L	1.009	0.999–1.020	0.079
Creatinine, umol/L	1.003	1.000–1.007	0.085
FBG, mmol/L	1.212	0.978–1.501	0.079
HbA1c (%)	1.524	1.064–2.183	0.021
TG, mmol/L	1.099	0.845–1.430	0.481
TC, mmol/L	1.169	0.774–1.764	0.458
HDL-C, mmol/L	0.887	0.611–1.288	0.528
LDL-C, mmol/L	1.071	0.829–1.384	0.598
LAAPD	0.989	0.966–1.013	0.377
LVAPD	1.014	0.975–1.055	0.476
IVS	1.000	0.880–1.136	0.997
LVPW	1.081	0.929–1.257	0.316
RVAPD	1.007	0.932–1.089	0.852
LVEF	1.008	0.972–1.046	0.654
caIMR	1.660	1.160–2.374	0.006

HR, hazard ratio; CI, confidence interval; caIMR, coronary angiography-derived index of microcirculatory resistance; DM, diabetes mellitus; PCI, percutaneous coronary intervention; ACEI/ARB, angiotensin-converting enzyme inhibitors/angiotensin receptor blockers; CCB, calcium channel blockers; β-R, beta-blockers; WBC, white blood cell; Hb, hemoglobin; FBG, fasting blood glucose; HbA1c, glycated hemoglobin; TC, total cholesterol; TG, triglycerides; HDL-C, high-density lipoprotein cholesterol; LDL-C, low-density lipoprotein cholesterol; LAAPD, left atrial anteroposterior diameter; LVAPD, left ventricular anteroposterior diameter; IVS, interventricular septum thickness; LVPW, left ventricular posterior wall thickness; RVAPD, right ventricular anteroposterior diameter; LVEF, left ventricular ejection fraction.

**FIGURE 3:**
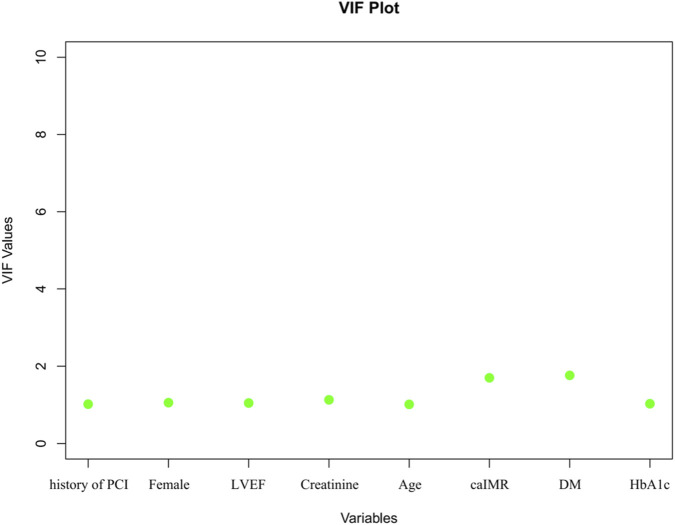
Variance inflation factor (VIF) analysis among DM, HBA1c, history of PCI, Female, LVEF, age Creatinine and caIMR. VIF, Variance inflation factor; HR, hazard ratio; CI, confidence interval; DM, diabetes mellitus; PCI, Percutaneous coronary intervention; HbA1c, glycated hemoglobin; LVEF, left ventricular ejection fraction; caIMR, coronary angiography-derived index of microcirculatory resistance.

To avoid potential multicollinearity, we assessed the variance inflation factor (VIF) for all candidate variables, including those with P < 0.05 in univariable analysis and the prespecified clinically relevant covariates (age, sex, LVEF, creatinine, and history of PCI). Variables with a VIF >2 were considered collinear and were excluded from the final multivariable Cox regression model (Figure 3). No variables exceeded the VIF threshold of 2; therefore, none were excluded from the final model.

To adjust for potential confounding, the multivariable Cox proportional hazards model was constructed by forcing in prespecified clinically relevant covariates, including age, sex, LVEF, creatinine, and history of PCI, regardless of their statistical significance in univariable analysis. In addition, variables demonstrating significant associations (P < 0.05) in univariable analysis were incorporated into the model following exclusion of collinear variables through Spearman correlation analysis (Table 3). The final model identified caIMR (adjusted HR = 1.547, 95%CI: 1.054–2.270; P = 0.026) as independent predictors of MACE in elderly patients with UA following PCI.

**TABLE 3:** Results of multivariable cox regression analysis of MACE.

Variables	HR	95%CI	P
Age, years	0.993	0.962–1.024	0.636
Female, n (%)	1.059	0.732–1.530	0.762
DM	1.505	0.939–2.413	0.090
History of PCI	1.146	0.781–1.682	0.486
Creatinine, umol/L	1.002	0.998–1.006	0.312
HBA1c	0.979	0.802–1.194	0.832
LVEF	1.009	0.972–1.047	0.641
caIMR	1.547	1.054–2.270	0.026

HR, hazard ratio; CI, confidence interval; DM, diabetes mellitus; PCI, percutaneous coronary intervention; HbA1c, glycated hemoglobin; LVEF, left ventricular ejection fraction; caIMR, coronary angiography-derived index of microcirculatory resistance.

### Subgroup analysis

**FIGURE 4:**
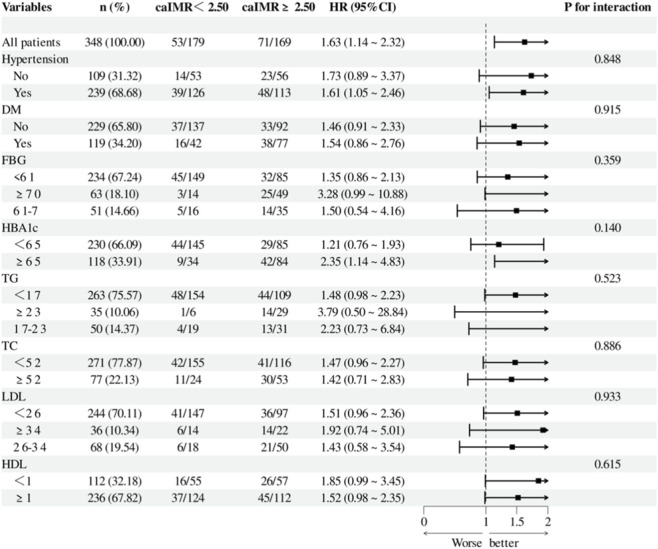
Subgroup analysis of correlation between high caIMR. caIMR, coronary angiography-derived index of microcirculatory resistance; DM, diabetes mellitus; FBG, fasting blood glucose; HbA1c, glycated hemoglobin; TC, total cholesterol; TG, triglycerides; HDL-C, high-density lipoprotein cholesterol; LDL-C, low-density lipoprotein cholesterol; HR, hazard ratio; CI, confidence interval.

The subgroup analysis demonstrated consistent prognostic implications of caIMR across diverse patient populations when using 2.50 mmHg s/cm as the cutoff value. Among all enrolled patients (n = 348, 100.0%), 53 events occurred in those with caIMR <2.50 mmHg s/cm, while 71 events were observed in patients with caIMR ≥2.50 mmHg s/cm, yielding a HR of 1.63 (95% CI: 1.14–2.32). The stratified analyses revealed comparable risk associations in patients with (HR = 1.61) and without (HR = 1.73) hypertension (P for interaction = 0.848). Similarly, diabetic and non-diabetic patients showed consistent risk patterns (HR = 1.54 vs. 1.46, respectively; P for interaction = 0.915). For analyses stratified by metabolic parameters—including FBG, HbA1c, TG, TC, LDL-C, and HDL-C levels—several strata had small event numbers (e.g., TG ≥ 2.3: n = 35, HR = 3.79, 95% CI: 0.50–28.84; FBG ≥7.0: n = 63, HR = 3.28, 95% CI: 0.99–10.88), resulting in wide confidence intervals. These results should be interpreted with caution, and the non-significant interaction terms (all P for interaction >0.05) do not definitively establish effect homogeneity across these underpowered subgroups (Figure 4). Collectively, these findings suggest that the prognostic impact of caIMR remains relatively uniform across clinically relevant patient subgroups, supporting its robust predictive value independent of conventional cardiovascular risk factors.

## Discussion

In the present study, we demonstrated that post-PCI caIMR ≥25 mmHg s/dm is an independent predictor of MACE in elderly patients with unstable angina undergoing PCI. The diagnostic threshold of caIMR ≥25 mmHg s/dm was adopted from the prospective, multicenter FLASH IMR study (Wang et al., 2025; Huang et al., 2023), which validated that a caIMR ≥25 mmHg s/dm, calculated using the same FlashAngio software, has excellent diagnostic accuracy for identifying coronary microvascular dysfunction, using the invasive wire-based index of microcirculatory resistance (IMR) as the gold standard.

However, comparison with recent studies—including the EARLY-MYO-AMR study—reveals that studies in patients with ST-segment elevation myocardial infarction (STEMI) have proposed higher optimal caIMR cutoffs for predicting adverse outcomes (Zhang et al., 2025; Li et al., 2024; Wen et al., 2024). Notably, the EARLY-MYO-AMR study by Zhang et al. — a large-scale, multicenter study involving 2,663 STEMI patients—identified an optimal AMR cutoff of >26.6 mmHg s/dm for predicting CMD diagnosed by cardiac magnetic resonance (CMR), with an AUC of 0.721 (Zhang et al., 2025). These findings strongly support the prognostic value of angiography-derived microcirculatory resistance indices in STEMI patients. These higher thresholds reflect the severe ischemia-reperfusion injury, distal atherothrombotic embolization, and microvascular obstruction characteristic of acute myocardial infarction. In contrast, our study enrolled elderly patients with UA, who typically have less severe microvascular injury. Consistent with this pathophysiological difference, the optimal caIMR cutoff in our cohort was lower and set at caIMR ≥25 mmHg s/dm (equivalent to 2.5 mmHg s/cm in the FlashAngio system). Notably, the EARLY-MYO-AMR study reported a cutoff of >26.6 mmHg s/dm (approximately 2.66 mmHg s/cm after unit conversion), which is numerically very close to our cutoff of 2.5 mmHg s/cm. The minor difference is attributable to variations in software algorithms, scaling factors, or reference standards (CMR vs. invasive IMR) (Szekely et al., 2025; Nicolau et al., 2025). Both thresholds consistently identify patients at elevated risk for adverse outcomes.

The concept of “microvascular angina” was first proposed by Cannon and Epstein in 1988 to characterize cases with typical myocardial ischemia symptoms despite normal coronary angiography (Cannon and Epstein, 1988). In 2013, this condition was formally classified as a distinct subtype of coronary artery disease (Filippo et al., 2014). Numerous clinical studies have demonstrated that CMD is closely associated with major adverse cardiovascular events (Aldujeli et al., 2024; Dimitriadis et al., 2025; SACdA et al., 2023). Its pathophysiological mechanisms primarily include: abnormal coronary microvascular vasomotor function leading to inadequate myocardial perfusion, prolonged ischemia triggering myocardial cell apoptosis and interstitial fibrosis, which in turn results in left ventricular diastolic dysfunction and even heart failure (Aldujeli et al., 2024; Gurgoglione et al., 2025). Additionally, coronary microvascular dysfunction can accelerate the progression of epicardial coronary atherosclerosis, thereby increasing the likelihood of myocardial infarction in patients (Campbell et al., 2024). In terms of diagnosis, the 2023 Chinese Expert Consensus systematically reviewed the diagnostic methods for CMD, with wire-based IMR being recognized as a convenient and reliable tool for assessing coronary microvascular status in the catheterization laboratory (Chen et al., 2023). In patients with acute coronary syndrome, IMR measured during direct PCI can reliably predict adverse events (Gregor et al., 2017; Prajith et al., 2024). Despite the growing body of research evidence supporting IMR, its invasive nature limits its clinical application. In contrast, caIMR is an emerging non-invasive detection technique that can rapidly and cost-effectively quantify microvascular function. Its diagnostic value has been validated in multiple clinical studies, including the FLASH IMR study and the SPECT-MPI validation study, confirming its promising clinical application prospects (Huang et al., 2023).

In alignment with existing literature, this study defined caIMR ≥25 as the diagnostic threshold for CMD, a cutoff previously validated to correlate with adverse outcomes in CAD patients (Zhou et al., 2025). Notably, evidence regarding caIMR’s prognostic utility in elderly UA patients post-PCI remains scarce. Our longitudinal analysis demonstrated significantly higher MACE rates in high caIMR (≥25 mmHg s/dm) *versus* low caIMR patients (P < 0.05), with multivariable Cox regression confirming post-PCI elevated caIMR as an independent MACE predictor (adjusted HR = 1.547, 95%CI 1.054–2.270). These findings extend prior STEMI cohort evidence, broadening caIMR’s prognostic applicability (Zhou et al., 2025). To further assess the clinical significance of high caIMR after PCI, we conducted a systematic subgroup analysis. The results showed that high caIMR was significantly associated with poor outcomes regardless of whether patients had hypertension (interaction P = 0.915) or diabetes (interaction P = 0.359), suggesting that its predictive value has broad applicability. Regarding metabolic parameters, the association was more pronounced in patients with impaired glucose metabolism: the HR in the HbA1c ≥ 6.5% subgroup was 2.35 (95% CI: 1.14–4.83), which aligns with previous reports that poor glycemic control is closely associated with an increased risk of microvascular and macrovascular complications (Bahrami Hezaveh et al., 2025). In recent years, novel therapeutic strategies aimed at improving microcirculatory function have garnered significant attention. Preliminary reports from the DAPAHEART trial suggest that dapagliflozin may enhance myocardial blood flow reserve by improving glucose metabolism (Leccisotti et al., 2022). As highlighted by Esdaile et al., intensive glycemic monitoring in post-MI diabetics may unveil novel therapeutic windows (Esdaile et al., 2023). Pathophysiologically, hyperglycemia-induced endothelial dysfunction *via* inflammatory pathways (e.g., CCL7 activation) provides a plausible mechanistic link between dysglycemia and CMD (C et al., 2024), though detailed mechanisms warrant further investigation.

This study suggests that caIMR may serve as a reliable prognostic marker for MACE in elderly patients with unstable angina undergoing PCI. The robust association between elevated caIMR and post-PCI outcomes underscores its potential as a risk stratification tool, enabling identification of high-risk populations who may benefit from intensified management strategies. However, these findings are hypothesis-generating, and future large-scale prospective studies are needed to validate the clinical utility of caIMR before routine application can be recommended. Thus, implementation of caIMR assessment in clinical practice remains an area warranting further investigation.

The findings of this study should be interpreted in light of several limitations. First, as a single-center retrospective study, detailed lesion characteristics, procedural details, acute cardiac function status, and granular glycemic control data were not available, which may have introduced residual confounding. Second, the sample size was modest, and findings require validation in larger prospective cohorts. Third, the caIMR cutoff was adopted from prior studies rather than derived from our own data. Fourth, the median follow-up of 27 months may be insufficient to capture late MACE events. Fifth, discrimination metrics (C-index, time-dependent AUC, NRI, IDI) were not calculated due to the exploratory nature of this hypothesis-generating study. Future large-scale prospective studies are needed to confirm and extend these findings.

## Conclusion

The caIMR demonstrated a significant positive correlation with the incidence of MACE. Multivariable Cox proportional hazards regression analysis confirmed caIMR as an independent risk factor for MACE. Notably, when applying the cutoff value of 2.50, the prognostic value of caIMR remained consistent across all clinically relevant subgroups, as evidenced by non-significant interaction terms.

## Data Availability

The raw data supporting the conclusions of this article will be made available by the authors, without undue reservation.
